# Endogenous endophthalmitis secondary to Lemierre’s Syndrome originating from pharyngotonsillitis

**DOI:** 10.1186/s12348-024-00420-2

**Published:** 2024-08-16

**Authors:** Nerea Gangoitia Gorrotxategi, Iñigo Salmeron Garmendia, Henar Heras-Mulero, Santiago López Arbués, Esther Compains Silva

**Affiliations:** Hospital Universitario de Navarra, Pamplona, Spain

**Keywords:** Lemierre’s syndrome, Endogenous endophthalmitis, Thrombophlebitis

## Abstract

**Purpose:**

The purpose of this article is to report a case of Lemierre’s Syndrome producing unilateral endogenous endophthalmitis in a healthy, young woman with a history of tonsillitis.

**Case report/observations:**

A 17-year-old healthy woman developed fever after a few days of sore throat. She later developed pneumonia with septic signs, leading to admission to the Intensive Care Unit. Lemierre Syndrome was diagnosed due to multiple septic pulmonary emboli and signs of sepsis following a recent episode of tonsillitis. During hospitalization, the patient complained of decreased visual acuity and floaters in her left eye. Ophthalmological examination revealed papillary edema, vitritis, foci of chorioretinitis in the macula and Roth’s spots, confirming the diagnosis of endogenous endophthalmitis. Subsequently, she underwent appropriate treatment, progressing satisfactorily.

**Conclusion and importance:**

Although ophthalmological manifestations are rare, due to the pathophysiological characteristics of Lemierre’s Syndrome, all patients should underwent standard ophthalmologic assessment, even in the absence of ophthalmic symptoms or visible findings, as part of a multidisciplinary management approach.

## Introduction

Lemierre’s Syndrome is characterized by infectious thrombophlebitis, typically affecting the internal jugular vein (IJV), resulting in multiorgan involvement, with *Fusobacterium Necrophorum* considered as the most associated organism [1]. Ophthalmic complications of Lemierre’s Syndrome have rarely been reported in the literature [2]. In this report, a case of an endogenous endophthalmitis in the context of Lemierre’s Syndrome complicated by *Streptococcus Anginosus* septicemia is described.

## Case report

**Fig. 1:**
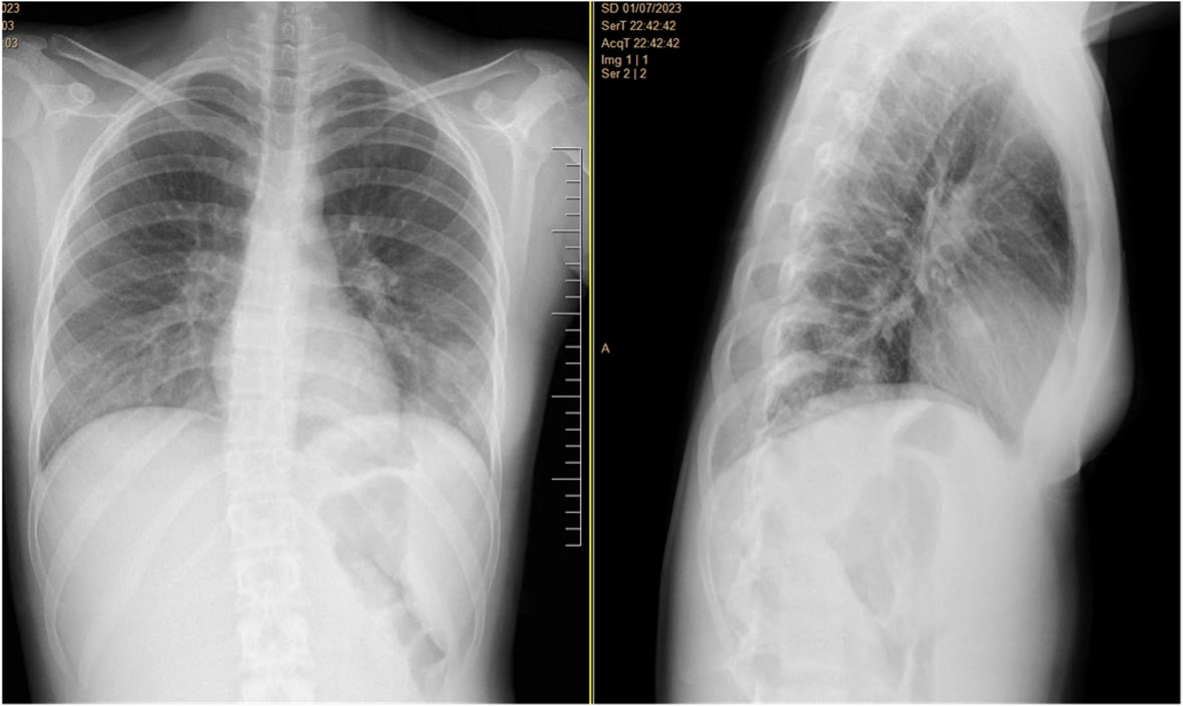
Chest X-Ray: consolidation focus in the left lower lobe of the lung

**Fig. 2:**
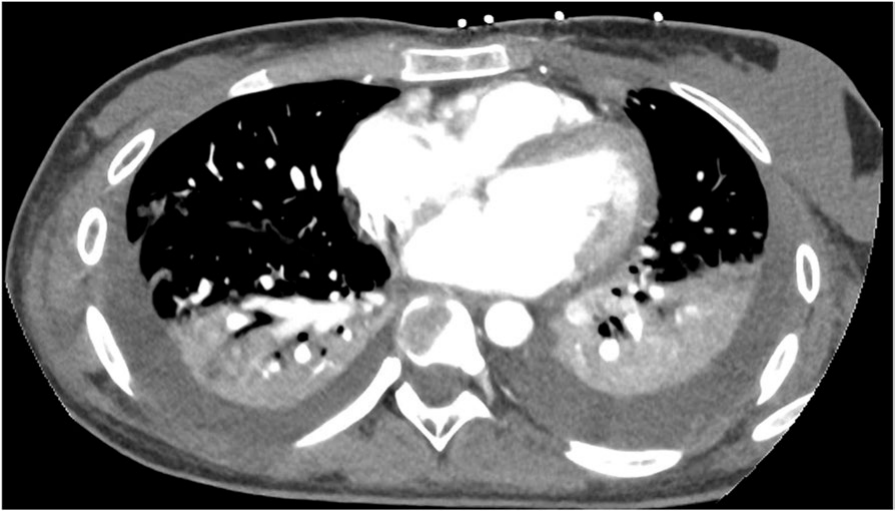
Toracic CT scan: hypodense nodular lesions, related to septic emboli. Moderate pleural effusion

**Fig. 3:**
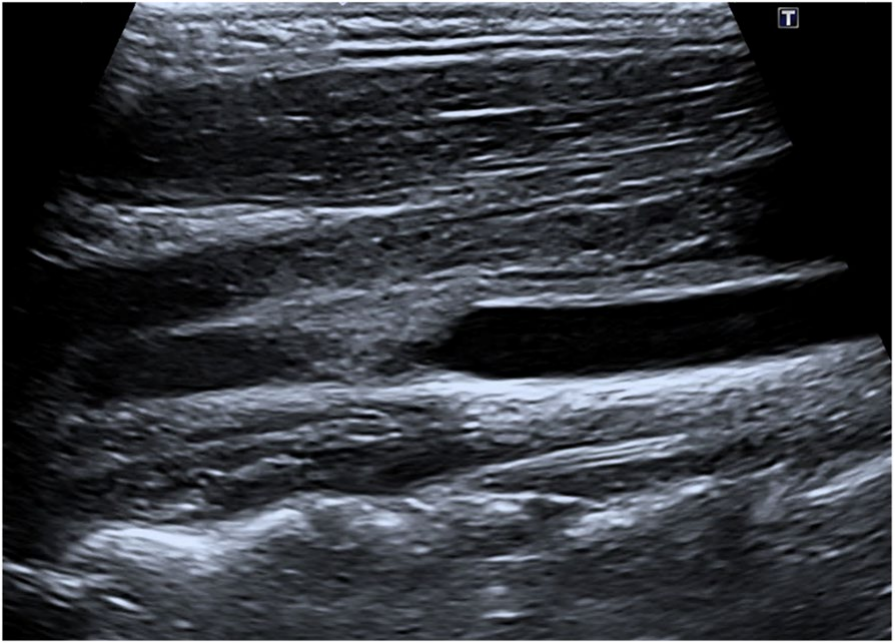
Ultrasound-jugular venous system: partial filling by echogenic material, compatible with thrombosis

**Fig. 4:**
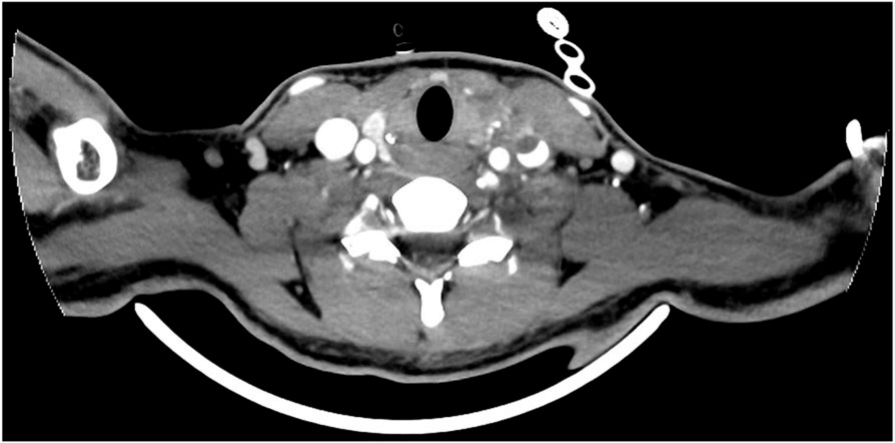
Cervical CT scan: partial filling of the left IJV, confirming thrombosis

A previously healthy 17-year-old woman, with a history of recurrent anginal illness, developed fever in the context of primary viral tonsillitis, which did not require antibiotics. One week later, the patient presented to the emergency department with chest pain, palpitations and cold sweats. A chest x-ray revealed pneumonia developing sepsis (Figs. 1 and 2). Patient was transferred to the intensive care unit (ICU) and started empirical treatment with intravenous Cefotaxime 2000 mg every 6 h and Levofloxacin 500 mg every 12 h, pending further examination. Subsequently, a cervical ultrasonography revealed partial occupation of the left IJV, consistent with thrombosis, confirmed by cervical Computed Tomography scan (CT scan) (Figs. 3 and 4). Pulmonary septic emboli were observed in the thoracic CT scan, confirming the diagnosis of Lemierre's Syndrome. Furthermore, *Streptococcus Anginosus* was isolated in blood cultures, leading to a switch from empirical antibiotic treatment to directed therapy with Ampicillin 12 g intravenously every 24 h and Clindamycin 600 mg intravenously every 6 h.

**Fig. 5:**
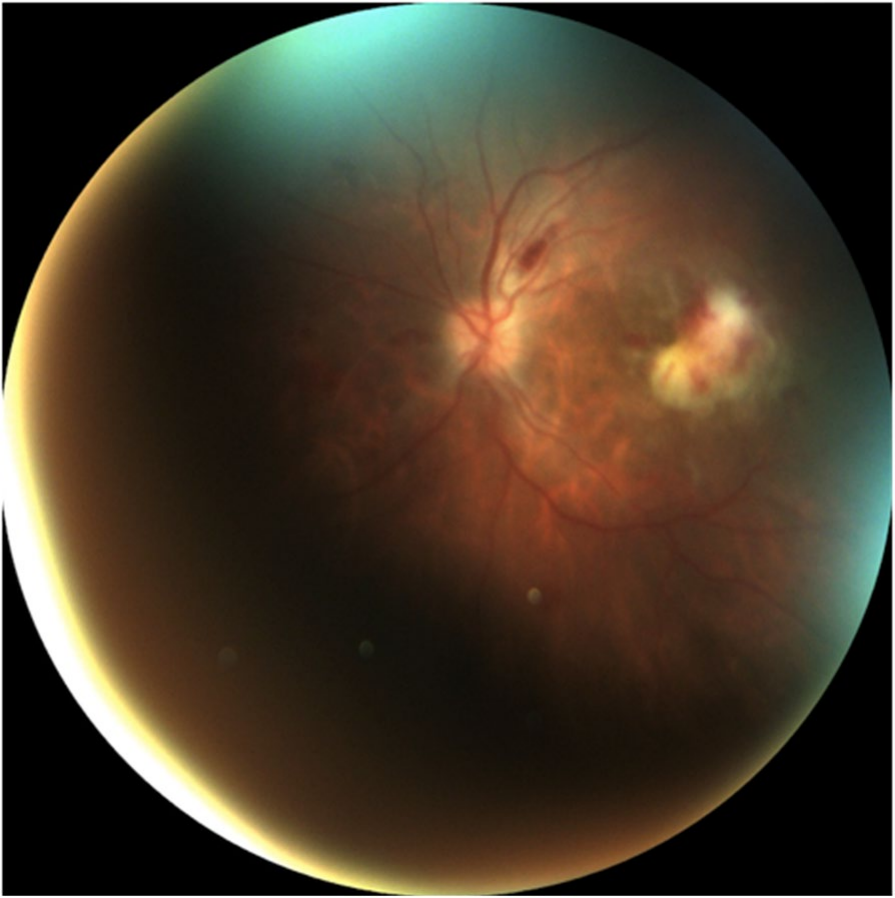
Left eye fundoscopy: papilledema, vitritis, chorioretinitis foci in macula and Roth spots. *Day 1*

While on systemic treatment (antibiotics, anticoagulation, analgesia, transfusion…), the patient reported decreased visual acuity with floaters in the left eye. Initial ophthalmologic examination revealed visual acuity of 20/20 in the right eye and 20/200 in the left eye. Indirect ophthalmoscopy showed papillary edema, vitritis, a whitish lesion with haemorrhagic component with the appearance of a focus of retinitis at the level of the macula and Roth's spots in her left eye (Fig. 5). Given the fundoscopic appearance of the lesion, considering a focus of *Streptococcus Anginosus* but unable to rule out Cytomegalovirus (CMV) retinitis, also considering the history of viral tonsillitis, it was decided to administer intravitreal Ganciclovir and Vancomycin (a total of 2 times each) and intravenous Ganciclovir 300 mg every 12 h. However, the ophthalmologic examination was limited due to the patient’s hospitalization in the ICU, hence, it was not possible to carry out vitreous sampling for cultures.

**Fig. 6:**
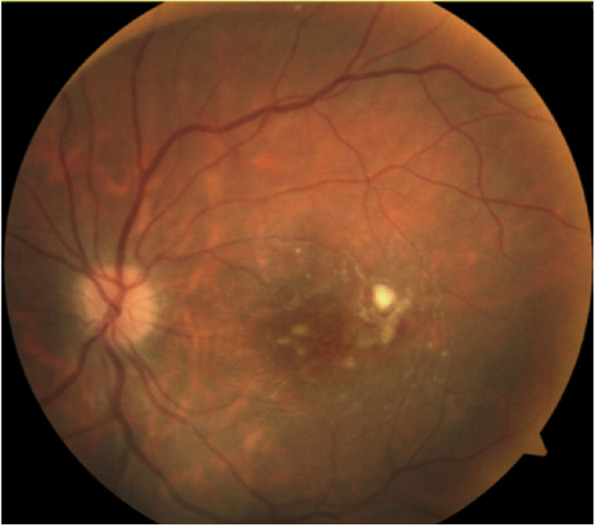
Fundoscopy: Smaller whitish lesion and less exudation area, no vitritis.* Day 10*

**Fig. 7:**
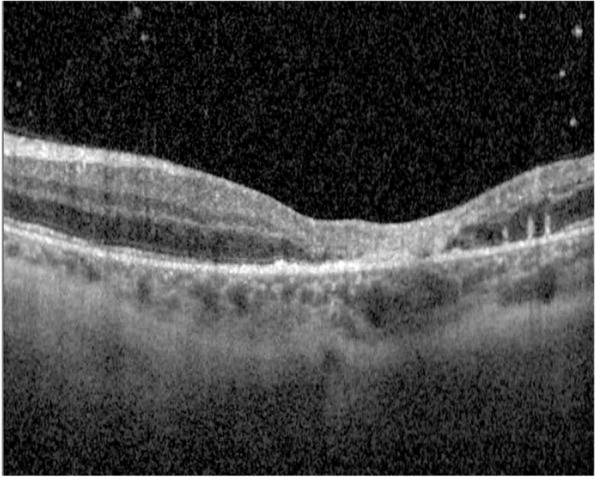
Optical Coherence Tomography (OCT): macular atrophy, with some cells in vitreous and choroidal thickening. *Day 10*

Initially, the patient showed favorable progress with a progressive decrease in vitritis and the whitish lesion temporally to the macula (Figs. 6 and 7). However, after negative Polymerase Chain Reaction (PCR) for CMV in blood, Ganciclovir was discontinued.

**Fig. 8:**
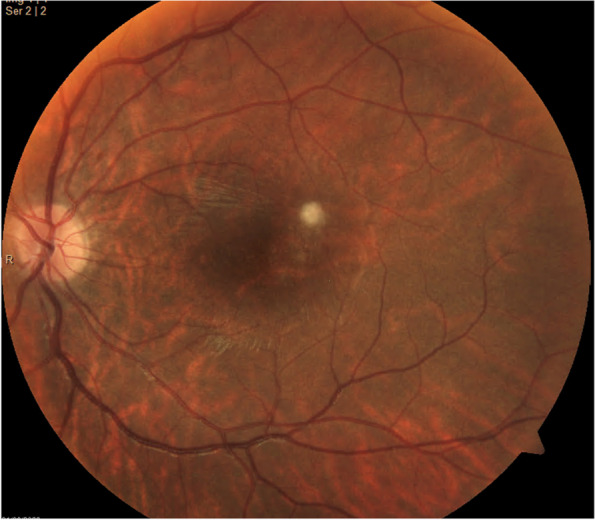
Fundoscopy: macular atrophy and a raised whitish area temporal to macula, without vitritis. *Month 1*

**Fig. 9:**
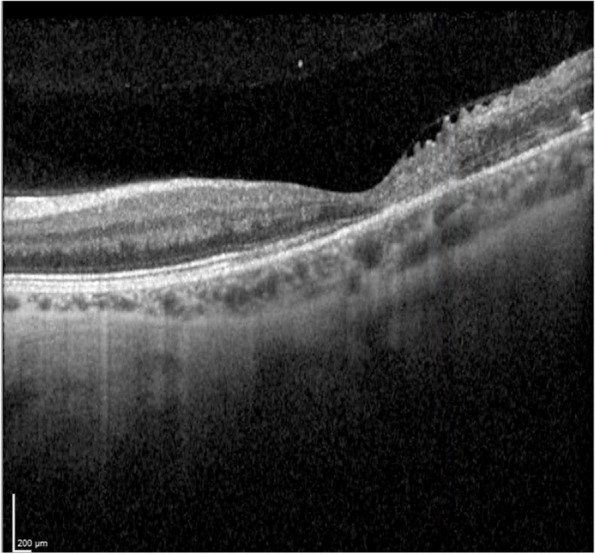
OCT: growth of fibrous appearance from Bruch’s to vitreous, with retinal disorganization. *Month 1*

**Fig. 10:**
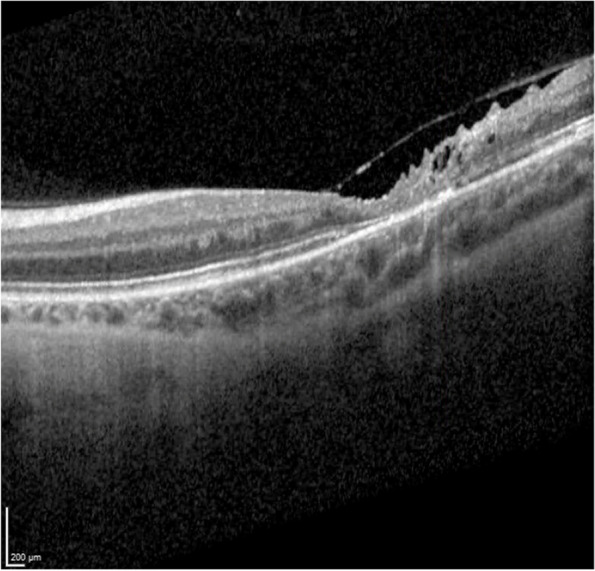
OCT: macular atrophy, without further retinal seeding. *Month 3*

Three weeks after the patient's initial presentation, the patient was discharged with satisfactory progress, visual acuity in patient’s left eye was 20/200, optical coherence tomography (OCT) demonstrating macular atrophy and retinal disorganization, with no further retinal lesions (Figs. 8 and 9). At the 3-month visit, the patient's vision remained stable, and the right eye was not affected at any time (Fig. 10).

## Discussion

Lemierre’s Syndrome was first described in 1936 by Andre Lemierre, who published 20 cases of anaerobic septicemia and septic thrombophlebitis that lead to the death of 18 patients, while in the pre-antibiotic era [3, 4]. It is a life-threatening condition that most commonly affects previously healthy young adults. It is characterized by head/neck bacterial infection, classically originating from pharyngitis, but it has also been described following dental infections, sinusitis, cellulitis, septic thrombosis of cavernous sinus. Thrombosis of the IJV with septic embolic complications in a range of sites of distant organs, primarily the lungs, is associated with gram-negative septicemia [1, 4–7]. The most common anaerobic pathogen is *Fusobacterium Necrophorum* [1, 5]; other implicated pathogens are *Streptococcus*, *Staphylococcus* sensible or resistant to methicillin, *Klebsiella* Pneumoniae, *Eschereichia Coli*, *Bacillus Cereus*, *Listeria* or *Pseudomonas Aeruginosa *[1, 4, 7, 8].

Lemierre’s Syndrome may cause a wide range of ophthalmic complications [5]. In 2022, the most extensive analysis conducted on ophthalmic complications related to this pathology was published. Twenty-seven patients with ocular manifestations were analyzed from a large cohort of 712 cases of Lemierre’s Syndrome. The most prevalent symptoms and signs were periocular edema or eye swelling (38%) and decreased visual acuity (35%), followed by impaired eye movements/nerve palsy (28%) and proptosis (28%). The most frequent diagnoses explaining the ophthalmic complications were cerebral vein thrombosis (70%) and superior ophthalmic vein thrombosis (55%).

Kreuzpointner, R. et al. described only two cases of endogenous endophthalmitis related to Lemierre’s Syndrome [5, 9]. The first reported case of endogenous endophthalmitis was by MA Ahad et al. [9] The patient exhibited typical signs and symptoms of the syndrome, initially presenting oropharyngeal infection and empyema. *Fusobacterium Necrophorum* was isolated from blood cultures and the patient developed IJV thrombosis and metastatic bacterial endophthalmitis during the disease course. Ocular symptoms included decreased vision in the right eye and anterior uveitis, with a vitreous white mass observed during examination. Treatment involved topical steroids, Atropine and sensitive antibiotics, resulting in improved ocular condition within four days. The patient remained on systemic antibiotics for 8 weeks, leading to resolution of endophthalmitis. Notably, Kreuzpointner, R. et al. did not reference a second case [5]. Therefore, this article presents the third documented case of endogenous endophthalmitis related to Lemierre's Syndrome, contributing valuable insights to the scientific community.

Generally, endogenous endophthalmitis has a low prevalence (approximately 2–8% of all cases of endophthalmitis). Pathogenesis occurs when organisms reach the eye via the bloodstream and then cross the blood ocular barrier [10]. In case of Lemierre's Syndrome, it occurs from the entry of the causal organism through the oropharyngeal mucosa from trauma, inflammation, or tissue destruction and it spreads from an abscess through the deeper loose connective cervical tissues, via an hematogenous mechanism, or spreads via a lymphogenic mechanism in order to extend into the veins of the head or neck with the most frequent route being from the tonsil into the ipsilateral IJV. This will eventually activate platelets, the coagulation cascade and cause inflammation leading to thrombus formation and Lemierre’s syndrome’s progression. The spread of septic emboli from the IJV, or whichever vein the bacterium has seeded, can lead to the involvement of distant organs within the body [11]. In case of ophthalmic artery involvement due to sepsis, endogenous endophthalmitis may occur and worsen the ocular symptoms. Therefore, in the presence of worsening of the ocular symptoms, endogenous endophthalmitis should be promptly ruled out. While this complication is rare, its possibility implies that the rate of ophthalmic complications in patients with Lemierre’s syndrome might be even higher if these patients underwent standard ophthalmologic assessment even in the absence of ophthalmic symptoms or visible ophthalmic findings like chemosis, swelling and proptosis [5].

It is considered that 25% of endophthalmitis cases due to Lemierre’s Syndrome are initially misdiagnosed [10]. Hence, the importance of accurate diagnosis. As stated in the title of the article written by JC Davis in 2012 [12], the clinician may face the diagnostic dilemma in retinitis and endophthalmitis. In the present 17-year-old woman case, CMV retinitis was considered in the initial differential diagnosis based on the fundoscopic lesion, history of previous viral tonsillitis and limitations of further examination. Therefore, it was initially treated as such because it could not be ruled out with certainty. However, given the patient had negative CMV PCR results, CMV retinitis was deemed unlikely, and treatment targeted at this condition was discontinued [12].

CMV retinitis occurs in immunocompromised patients, such as those with HIV/AIDS, undergoing chemotherapy or receiving immunomodulators, often in the context of a systemic infection, characterized by full-thickness retinal necrosis that leaves a thin, atrophic and gliotic scar, potentially leading to retinal detachments as a common complication, often due to multiple retinal tears at the border of normal retina and the atrophic scar. Two clinical morphologic variants of CMV retinitis have been described: 1) fulminant or hemorrhagic, characterized by a more extensive area of retinal edema and necrosis, mixed with hemorrhage, often resembling a “pizza pie” or “cottage cheese and ketchup” appearance, occurring more frequently in the posterior pole; and 2) granular appearance, which affects more often in the periphery. For its diagnosis, CMV can be detected in the blood of the majority of patients with CMV retinitis either by culture or PCR of CMV from a blood specimen. In terms of the differential diagnosis [13, 14]. Treatments for CMV retinitis have included intravenous antivirals (Ganciclovir, Foscarnet, Cidofovir, Valganciclovir) and intravitreal injections of either Ganciclovir or Foscarnet. Many patients, especially those with lesions threatening the fovea or optic nerve, are treated with an initial series of intravitreal injections of either Ganciclovir or Foscarnet, combined with systemic therapy (e.g. Valganciclovir).

The presence of ophthalmic involvement may represent a signal of cerebral vein involvement requiring prompt action [5]. Treatment of Lemierre’s Syndrome revolves around aggressive antibiotic treatment, anticoagulation therapy and surgical management. Delaying antibiotic treatment may increase mortality and affect long-term morbidity [11]. Prior to antibiotics, the disease carried a mortality rate of 32–90%, compared to 5–18% with antibiotic treatment [1]. Consequently, initial treatment should include broad-spectrum antimicrobial therapy, which is subsequently narrowed once the culprit bacterium is identified. Endogenous endophthalmitis should be treated with systemic antibiotics, combined or not with intravitreal antibiotics. Intravitreal Vancomycin is the most commonly selected agent for Gram positive coverage and Ceftazidime the most commonly used antibiotic for Gram negative infection. Vitrectomy can also be performed, usually with the administration of intravitreal antibiotics and vitreous sampling for microscopy and culture [10].

The case presented in this report adds to the short list of endogenous endophthalmitis related to Lemierre’s Syndrome. This patient meets the diagnostic criteria for Lemierre’s Syndrome, which includes primary infection in the oropharynx, positive blood cultures for *Streptococcus Anginosus*, imaging evidence of internal jugular venous thrombophlebitis and lung metastatic focus [4]. She was promptly managed with broad-spectrum antimicrobial therapy, along with intravitreal antibiotics, which were adjusted once the culprit bacterium was identified and depending on the evolution of the fundoscopic lesion. The patient’s prompt reporting of ocular symptoms such as decreased visual acuity and floaters, as well as the close ophthalmological follow-up, allowed for the prevention of complications in the affected and fellow eyes, and even systemic ones that could compromise the patient’s life. The lack of adequate consciousness in some of these patients hospitalized in the ICU may underestimate the presence of ophthalmic complications associated with this pathology, potentially delaying timely diagnosis and treatment. Therefore, and as emphasized by Kreuzpointner, R. et al., there is a need for an interdisciplinary approach to the management of patients with Lemierre’s Syndrome, with a routine involvement of ophthalmologists [3, 5].

## Conclusion

Ophthalmological manifestations of Lemierre’s Syndrome are rare but severe, including thromboembolic complications, among which endogenous endophthalmitis is included. Multidisciplinary management is essential, necessitating very close ophthalmological follow-up, even in patients without ophthalmic symptoms. Early administration of both systemic and intraocular antibiotics should be the mainstay of treatment for most patients, as endophthalmitis can often lead to devastating ocular and systemic consequences.

## Data Availability

No datasets were generated or analysed during the current study.

## Abbreviations

IJV: Internal Jugular Vein
ICU: Intensive Care Unit
CMV: Cytomegalovirus
CT: Computed Tomography
PCR: Polymerase Chain Reaction
OCT: Optical Coherence Tomography
NGG: Nerea Gangoitia Gorrotxategi
HHM: Henar Heras-Mulero
SLA: Santiago López Arbués
ISG: Iñigo Salmeron Garmendia
ECS: Esther Compains Silva
